# Virginia Society of Surgeon Dentists

**Published:** 1845-03

**Authors:** George W. Munford

**Affiliations:** C. H. D.


*Virginia Society of Surgeon Dentists*—It will be seen by the following, that the Legislature of Virginia have incorporated the above named association.

[*Eds.*

*An act Incorporating the Virginia Society of Surgeon Dentists.*

[Passed February 3d, 1845.]

Whereas, Samuel Lethbridge, John G. Wayt, James D. McCabe, and others, citizens of the State of Virginia, have formed themselves into a society, under the title of "The Virginia Society of Surgeon Dentists," whose objects are the promotion of dental science, the establishment of a library and cabinet of anatomical preparations, for the purpose of introducing a sound system of dental education: and it having been represented to the General Assembly, that the members of said society are desirous of obtaining a charter of incorporation, that they may with lawful protection carry out their laudable purposes:

*Be it therefore enacted*, That the members of the aforesaid society, together with such others as shall hereafter be associated with them, and their successors, are hereby created, a body politic and corporate, by the name and style of the "Virginia Society of Surgeon Dentists," and by that name shall have perpetual succession, and a common seal, and may hold and dispose of lands and tenements, goods and chattels, "not exceeding twenty-five thousand dollars at any one time," and may sue and be sued, plead and be impleaded.

The said society may also enact such constitution and by-laws, ("not repugnant to the laws of this state, and of the United States,") as they may think proper, for carrying into effect the object of the institution. And may do all other acts, proper for corporate bodies to do.

The legislature reserves the right to modify or repeal this act.

This act shall be in force from the passing thereof.

I certify that the foregoing is a true copy.

*Feb.* 7, 1845. GEORGE W. MUNFORD, C. H. D.

